# Mapping survey of schistosomiasis and soil-transmitted helminthiases towards mass drug administration in The Gambia

**DOI:** 10.1371/journal.pntd.0009462

**Published:** 2021-07-22

**Authors:** Yaya Camara, Bakary Sanneh, Ebrima Joof, Abdoulie M. Sanyang, Sana M. Sambou, Alhagie Papa Sey, Fatou O. Sowe, Amadou Woury Jallow, Balla Jatta, Sharmila Lareef-Jah, Sainey Sanneh, Flobert Njiokou, Abdoulie Jack, Serign Jawo Ceesay, Chinyere Ukaga

**Affiliations:** 1 Epidemiology and Disease Control Department, Ministry of Health and Social Welfare, Kotu, The Gambia; 2 National Public Health Laboratories, Ministry of Health and Social Welfare, Kotu, The Gambia; 3 Health Management Information System, Ministry of Health and Social Welfare, Kotu, The Gambia; 4 World Health Organisation technical support team; The Gambia Country Office, Inter-country Support Team, Bobo-Dioulasso, Burkina Faso; 5 Health Research Directorate, Ministry of Health and Social Welfare, Kotu, The Gambia; 6 Project Consultant (Retired), "Tranquil", West Coast Region, The Gambia; 7 Medical Research Council Laboratories, Bakau, The Gambia; Seoul National University College of Medicine, REPUBLIC OF KOREA

## Abstract

**Background:**

A national mapping survey of schistosomiasis (SCH) and soil-transmitted helminthiases (STH) was conducted in The Gambia in May, 2015. The survey aimed at establishing endemicity of schistosomiasis and soil-transmitted helminthiases to inform decisions on program planning and implementation of mass drug administration (MDA).

**Methodology/Principal findings:**

A cross-section of 10,434 eligible school aged children (SAC), aged 7 to 14 years old were enrolled in the survey. The participants were randomly sampled from 209 schools countrywide using N/50, where N = total eligible children per school. Stool, and urine samples were provided by each child and examined for schistosomiasis and soil-transmitted helminthic infections using double Kato-Katz, urine filtration, dipstick techniques and CCA rapid test kits. Data were managed using online LINKS system enabling real-time data availability and access. Epi Info version 3.5.3 and health mapper version 4.3.2 were used to generate outputs of endemicity and distribution. Descriptions of mapped districts for MDA eligibility and frequency were done with reference to WHO PC strategy recommendations. Mapping results indicated that nationally, the prevalence of schistosomiasis (SCH) and soil-transmitted helminthiases (STH) was 4.3% and 2.5% respectively. In terms of distribution STH are more common in Western Region One (WR1) at 4.1% prevalence, then Lower River Region (LRR) 3.6%, and Western Region Two (WR2) 3.0%. In contrast, SCH indicated much higher prevalence in Central River Region (CRR) at a rate of 14.2%. This is within medium prevalence range, and is followed by Upper River Region (URR) at 9.4%, which is within low prevalence range. At the district level, schistosomiasis prevalence seems to be highest in Niani district (22%) in CRR. Banjul island, the capital city, seems to have the highest prevalence of STH (up to 55%), followed by Kombo South with 22% prevalence. *Schistosoma haematobium* characterised by haematuria, was the most dominant infection of schistosomiasis discovered followed by *Schistosoma mansoni* which reported in 0.1% of infections. Out of 42 districts mapped 14, or 38%, of them are co-endemic for soil-transmitted helminthiases *(*ascariasis, trichuriasis, and hook-worm infections) and schistosomiasis *(S*. *haematobium* and *S*. *mansoni)*.

**Conclusions:**

We identified that 24/42(57%) districts mapped in The Gambia are endemic for schistosomiasis expressing the need for preventive chemotherapy. Twenty (47%) of the districts mapped are endemic for STH. However, only two STH endemic districts namely Banjul (55%) and Kombo South (22%) were within rates eligible for mass drug administration.

## Author summary

The Gambia joined the international community in the fight against neglected tropical diseases (NTDs), in order to enable control and eventual elimination by year 2020. This goal is in line with Africa regional targets for NTDs control and elimination. This decision was the driving force for a tabletop analysis of the existing NTD situation in the country, culminating in the development of a comprehensive five-year NTD Master plan 2015–2020, and an Annual Work Plan 2016. The World Health Organisation regional office for Africa’s Regional Program Review Group (WHO/AFRO RPRG) supported this work by granting approval for the mapping of schistosomiasis (SCH) and soil-transmitted helminthiases (STHs) across the country in May 2015. In this publication, we describe the purpose of the survey, how the survey was conducted, and how data and specimens were collected, handled, analyzed and interpreted. We have discovered that schistosomiasis exists in twenty-four districts in the country. Most of these districts are located in the eastern half of the country in Upper and Central River Regions. This means that communities in the affected districts require treatment to reduce the burden of disease. Treatment for schistosomiasis is unlike soil-transmitted helminthiases because all districts with schistosomiasis are eligible for treatment irrespective of the level of prevalence and endemicity according to the guidelines of the World Health Organisation (WHO). In comparison to STH, mass drug administration is not required for low STH endemic communities where the prevalence was less than 20%. Instead such communities will benefit from case by case treatment STH. Mass treatment will be required in district where STH prevalence was at least moderate or 20% and above. Soil-transmitted helminthiases were found in twenty districts. However, it is important to highlight that only two of these districts, with prevalence of 22% and 55%, respectively were eligible for mass drug administration in accordance with WHO guidelines. The guidelines recommend that districts with a prevalence of soil-transmitted helminthiases of less than 20% do not require mass community treatment in order to achieve disease elimination.

## Introduction

Globally, one billion people are estimated to be at risk of schistosomiasis (SCH), soil-transmitted helminthiases (STH) and other neglected tropical diseases (NTDs), and approximately 534,000 people die of the diseases annually [1]. Neglected tropical diseases are widespread in Africa, and are the most common cause of infections in people living in poverty globally [2–4]. In sub-Saharan Africa, NTDs are high in prevalence accounting for approximately one-quarter to one-third of global cases of the three major intestinal helminth infections; ascariasis, trichuriasis, and hookworm infection and all or most of schistosomiasis infections, such as those due to haematobium, mansoni, japonicum and intercalatum [5,6]. In The Gambia, schistosomiasis (SCH) and soil-transmitted helminthiases (STH) have been known to be endemic as far back as the 1950s. In 2007, efforts to combat Neglected Tropical Diseases globally, were redirected to shared commitments to support the strategies, goals and targets by the World Health Organisation, after the first Global Partners Meeting [7]. This move resulted in a tremendous gain for public health, including scaling up of control and elimination programmes, by improving access to chemotherapeutic interventions for hundreds of millions of poor and marginalized individuals, in an innovative and cost-effective way [8]. Subsequently, WHO in 2012, developed a road map towards reduction of NTD related mortality and morbidity by 2020 [8].

In any NTD affected country, knowledge of local endemicity is essential for the development of appropriate intervention strategies such as preventive chemotherapy/mass drug administration (MDA), and integration of Water, Sanitation and Hygiene (WASH) projects [9].

NTDs of historic concern to The Gambia included lymphatic filiariasis, also known as elephantiasis; that is now successfully eliminated [10]. Whereas currently, both schistosomiasis, and soil-transmitted helminthiases remain endemic. Trachoma and leprosy are currently at various phases of pre-elimination. The focus of this study is on schistosomiasis and soil-transmitted helminthiases, which are the two known endemic Preventive Chemotherapy (PC) NTDs in the country. Urinary schistosomiasis probably existed in The Gambia for a great number of years. However, it was not until the general survey conducted by Jones and Thomas in 1945, and the observations of Thomas in 1947, and Ross in 1947, that the disease was shown to be endemic, with high incidence in the eastern regions of the country [11] such as Central and Upper River Regions.

Current data on the endemicity of urinary schistosomiasis (SCH) is lacking, with the limited evidence reported in the early 1950s to late 1980s [7,11,12], likely to be outdated. These studies focused mainly on known prevalence, pathogenesis, treatment and snail epidemiology. The dearth of current data made it a necessity to conduct mapping to determine occurrence of schistosomiasis (SCH) and soil-transmitted helminthiases (STH) in the country, to serve as the basis for the formulation of appropriate interventions [13–15]. A desk-based review was conducted in May 2014, as a follow up to examine reports of SCH and STH in The Gambia. The review exercise revealed an information gap on endemicity and co-endemicity to inform and guide evidence-based strategic intervention such as MDA [13]. The desk-based review gave rise to the development of an action plan for the national NTD focal point in December 2014, highlighting the need to conduct nationwide baseline mapping surveys in order to assess and establish the endemicity of SCH and STH. The main objective of the survey was to determine the endemicity status, the prevalence and spatial distribution of schistosomiasis and soil-transmitted helminthiases. This information would be invaluable in informing and guiding decisions on the implementation of interventions to control and eliminate these NTDs by 2020.

## Methods

### Ethics statement

Ethical approval was obtained from the Scientific Coordinating Committee (SCC 1419) of The Gambia government and MRC Joint Ethics Committee. Before the start of field work, field workers known as cluster monitors working in the Ministry of Education, who are responsible for monitoring and supervising educational institutions within and area called a cluster, worked with Regional Health Promotion Officers (RHPO) to visit and sensitize local authorities on the survey. They also conducted the identification of schools to be visited by the mapping teams. Heads of participating schools were provided with a written consent form to sign on behalf of the parents for the children to participate. Additionally, each survey team was provided with copies of the Information Education and Communication (IEC) materials to aid explanation of their mission to school children on each visit to a selected school, and it was explained to the children that participation was entirely voluntary.

### Study design and sampling

We observed a cross-section of 10,434 school-aged children who were randomly selected from 209 sampled schools countrywide. In each sampled school, 50 pupils, comprising of 25 boys and 25 girls, were selected through simple random sampling. In order to select eligible children from each row, a formula of N/50 (where N = total eligible pupils), was used to obtain the required 50 participants per school. A short sensitization on the procedures for urine and stool collection was conducted for the sampled children, each eligible child was then interviewed and provided two separate specimen containers, a pair of latex gloves, and a clean sheet of paper for urine and stool collection. As much as 5 ml of urine and approximately 5 to 10 grams of stool were requested for laboratory analysis.

### Sites selection

According to the WHO AFRO NTD mapping guide [14], five schools were identified for mapping within each district. By implication, this meant that mapping a total of 42 districts in The Gambia would yield 210 schools i.e. (42*5 = 210) when five schools are sampled in each district. We realised however that a number of districts had very few schools to sample according to the guidelines. The listing of all schools in the country by district revealed that some districts such as Foni-Bondali, and Niamina-Dankunku, had less than the required five schools needed for the survey. Consequently, Regions were used as the sampling unit instead of districts, although the total sample size of 210 schools required nationally for the survey remained the same. To achieve this, the sample size per region was determined as number of districts in a region multiplied by five. The total number of schools obtained through this procedure is used as the number of schools to be surveyed in that region. Finally, a random selection of schools was achieved using random number generation in Microsoft Excel.

### Sampling procedure used

The total number of schools per region was calculated as [**X**], total number of schools nationally was calculated as [**TX**], total number of schools to be selected per region was calculated as [X/TX * 210 = ns], figures were rounded up or down to the nearest whole number as [**ns**], and **5:** a random number table was used to select the exact number of schools as **‘n**^**s**^**’** for each region in a skip interval. Eventually a total of 209 lower basic schools were selected for the survey and the names of the selected schools were grouped in the respective districts of their location.

### Selection and enrolment of study participants; school-age children (SAC)

#### Eligibility criteria, inclusion and exclusion

All pupils within the age of 7–14 years enrolled in the sampled schools were eligible. Pupils not in the stated age range of 7–14 were excluded during the selection process in eligible schools.

### Selection procedure of study participants; school-age children

Within each school, the survey teams explained to the school authorities the objectives of the study and the procedures involved. After the students had been sensitized and they consented to be part of the survey, all the eligible children according to age and lack of ill health form two separate rows of boys and girls. Thereafter a random selection of participating children was carried out in accordance with the eligibility criteria stated above. In each school, 50 participants (25 boys and 25 girls) were randomly selected [14]. A contingency of up to 10 additional pupils; five boys and five girls were also selected in case any of the 50 sampled pupils failed to provide all the required specimens for laboratory analysis. Selected candidates were provided with a white sheet of paper to poo on and two plastic containers, one to produce urine and another with spatula fixed cap for scooping stool samples from the paper provided. To ensure hand hygiene after providing excrements, each child is also provided with a tissue paper, water and soap to clean their hands after specimen collection.

### Enrolment of study participants

After the collection of both urine and stool samples, the specimens were tagged with duplicate barcodes pasted onto the sides of the specimen containers. Data collectors enrolled the pupils by recording demographic information and risk of coming in contact with water bodies for each pupil using a smart phone. The bar code for each pupil was scanned or entered manually using the same phone as part of the questions on the individual questionnaire inbuilt on the phone.

### Laboratory investigation of specimens

The presence of infections of schistosomiasis and soil-transmitted helminthiases were examined through urine filtration, Kato-Katz and CCA techniques. Thus, the use of CCA was meant for making additional available through the study. Urine and stool specimens were screened for the presence of intestinal helminth and schistosome eggs with light microscopes and exposure with urine dipstick rapid diagnostic test method. Each urine sample was first examined for haematuria (blood in urine) using the dipstick and microscopy method. A test strip was dipped into a urine sample and the result read in 60 seconds by comparing against the manufacturer colour chart. Urine samples were gently stirred and 10 ml were filtered through a membrane using a syringe. The membrane filter was placed on a microscope slide and examined by a microscopist. Stool samples were examined for presence and intensity of *S*. *mansoni* and soil-transmitted helminthiases using the Kato-Katz technique. Two microscopists independently examined the smears, any discrepancies between their findings were discussed to decide upon the final result.

### Data management

Mapping data were collected both manually, using predesigned paper-based questionnaires, and electronically, using smart phones. The LINKS application software was used with an inbuilt questionnaire hosted online for Afro NTD mapping projects. Data collected from the field through LINKS was transmitted electronically and simultaneously using the internet connection of a mobile telephone network from any part of the country to a central server. Mapping data were therefore readily accessible online and in realtime to only a number of authorised users, including the national mapping data manager, NTD focal person and database administrator. In the absence of internet connection when data were collected in the field, data were stored on the phone and could be edited. Paper-based questionnaires were used to record laboratory data including the results of each tested specimen. This served as a reference for the verification of typographical errors on data transmitted electronically.

Data from the server were downloadable in excel file format for use. Downloaded datasets were cleaned using Microsoft excel 2010 version and tabulated. Further analysis of the cleaned data were done differently using Epi Info software version 3.5.3, and Health mapper software version 4.3.2, to run statistical outputs showing prevalence of both diseases nationally, regionally, by districts, by gender, and by specific parasites detected.

## Results

In Table 1, below we find that the number of children enrolled and tested concurrently for both soil-transmitted helminthiases and schistosomiasis were 10,434; with a mean age of 10.1 (± 2.1) years. Approximately 50.1% or 5, 230 pupils were male and the remaining 49.9% or 5, 204 were female. Those tested were grouped into age brackets of 7–9 years (4,498 [43.1%]), 10–12 years (4, 224 [40.5%]) and 13–14 years (1,712[16.4%]) respectively.

**Table 1:** Demography of study participants; schistosomiasis and soil-transmitted helminthiases -mapping survey in The Gambia.

Total School Age Children Tested		By Sex/Gender		By Age Categories		
		Female	Male	7 to 9 years	10 to 12 years	13 to 14 years
**National**	No Tested	n (%)	n (%)	n (%)	n (%)	n (%)
	10,434	5,204 (50%)	5,230 (50%)	4,498 (43%)	4,224 (40%)	1,712 (16%)
**By Region**						
**Central River Region**	1,915	955 (50%)	960 (50%)	937 (49%)	763 (40%)	215 (11%)
**Lower River Region**	1,100	550 (50%)	550 (50%)	453 (41%)	432 (39%)	215 (20%)
**North Bank East Region**	1,157	573 (50%)	584 (50%)	490 (42%)	437 (38%)	230 (20%)
**North Bank West Region**	607	299 (49%)	308 (51%)	283 (47%)	204 (34%)	120 (20%)
**Upper River Region**	1,451	723 (50%)	728 (50%)	750 (52%)	509 (35%)	192 (13%)
**Western Region 1**	2,551	1,276 (50%)	1,275 (50%)	999 (39%)	1,135 (44%)	417 (16%)
**Western Region 2**	1,653	828 (50%)	825 (50%)	586 (35%)	744 (45%)	323 (20%)

Key: *% = percentage, *n = absolute number.

The national prevalence of both soil-transmitted helminthiases and schistosomiasis was found to be low; 2.5%, and 4.3% respectively. In terms of geographical distribution, soil-transmitted helminthiases were more common in three of the seven health regions on the western half of the south bank of the River Gambia. Western Region1 registered 4.1% prevalence of STH, 3.6% in Lower River Region and 2 3.0% in Western Region2 respectively. Schistosomiasis, also known as Bilharziasis, was found to have highest prevalence in Central River Region at 14.2%, which is within medium prevalence range, followed by Upper River Region at 9.4%, which is within the range of low prevalence.

Schistosomiasis, which is caused by the parasitic fluke called *S*. *haematobium*, was the predominant type across the country. It was found to be most prevalent in the Central River Region reaching 14.8% prevalence, followed by Upper River Region with 9.5% prevalence. The prevalence in Western Region was virtually negligible at only 0.6%. North Bank East Region was the only region where no positive case of schistosomiasis was detected.

In contrast to schistosomiasis, soil-transmitted helminthiases were most prevalent in Western Region with a prevalence of 4.6%. When analysed as individual helminths, it was discovered that *Trichuris trichiura* was only 0.1% prevalent, making it the least prevalent of the STHs found in the country. Other STH infection due to hookworm and *Ascaris* were 0.6% and 1.8% respectively at the national level. At regional level, Western Region bore the largest burden of *T*. *trichiura at* 0.5%, followed by URR, which registered barely 0.1%. The above information is presented in Table 2, see below.

**Table 2:** Prevalence of Parasitic infection (%) among a cross section of school age children of 7–14 years across all Regions of The Gambia in May, 2015.

	#of People Tested	S. haematobium	S. mansoni	Total SCH	A. lumbricoides	Hookworm	T. trichuria
National	10434	4.2%	0.1%	4.3%	1.8%	0.6%	0.1%
Central River Region	1915	14.2%	0.4%	14.6%	0.6%	0.5%	0.0%
Lower River Region	1100	1.1%	0.0%	1.1%	3.1%	0.5%	0.0%
North Bank East Region	1157	0.0%	0.0%	0.5%	0.6%	0.2%	0.0%
North Bank West Region	607	1.5%	0.0%	1.5%	0.2%	0.0%	0.0%
Upper River Region	1451	9.4%	0.1%	9.5%	1.5%	0.2%	0.1%
Western Region 1	2551	0.0%	0.0%	0.0%	4.0%	0.4%	0.5%
Western Region 2	1653	0.6%	0.0%	0.6%	60.0%	2.0%	0.0%
By Gender							
Female	5204	3.7%	0.1%	3.8%	2.1%	0.6%	0.2%
Male	5230	4.7%	0.1%	4.8%	1.5%	0.6%	0.1%

Key: *% = percentage, *# = number, *SCH = Schistosomiasis, *S = Schistosoma, *A = Ascaris, *T = Trichuris.

Survey data revealed that 38%, or 14 districts in The Gambia, are STH and SCH co-endemic. Twenty-one districts, or 50%, of districts mapped in the country are endemic for STH. On the other hand, 18 districts, or 49% of the districts were endemic for SCH. Only one district had a high STH prevalence rate of 55%, thus requiring two annual treatment rounds with Albendazole [16]. Another district, called Kombo South, recorded a moderate prevalence of STH of 22%, and required only one round of mass treatment. The remaining 19 endemic districts with low STH prevalence required treatment on a case-by-case basis according to WHO guidelines that recommend MDA for STH control is necessary when parasite prevalence exceeds 20% in the target population [16].

## Discussion

Schistosomiasis and soil-transmitted helminthiases were both found to be endemic in the country, however, there were notable contrasts in the distribution of each. Schistosomiasis was found most on the eastern part of the country, where there are numerous fresh water bodies fed by rains and the River Gambia. It is worth noting that this eastern part of the River Gambia, comprising the regions of Central and Upper River, remains fresh all year round thus providing suitable snail vector breeding sites. In contrast to the distribution of schistosomiasis, soil-transmitted helminthiases were found mostly in the western half of the country where more than half of the population of The Gambia resides. The Western part of the River Gambia is generally closest to the Atlantic Ocean where it empties, and remains salty throughout the year.

National prevalence of schistosomiasis and soil-transmitted helminthiases appeared to be 4.3% and 2.5% respectively. Urinary schistosomiasis caused by *S*. *haematobium*, which accounts for 4.2% prevalence is the most dominant parasitic infection of all the identified parasites nationally. It is most endemic in the Central River Region where prevalence appears to reach 14.2%, and Upper River Region with 9.4% prevalence. These findings of endemicity in the aforementioned regions are consistent with discoveries of earlier studies [17]. Similarly, *S*. *haematobium* infection accounted for the high burden of schistosomiasis in South Sudan [15]. In Upper and most parts of Central River Region, the River Gambia remains fresh throughout the year thus enabling harvesting of its water for the irrigation of rice and other crops. Moreover, seasonal pools abounding the laterite plateau in these two regions, as well as the existence of major irrigated rice fields such as Jahally-Pacharr rice fields and Kuntaur rice fields, contribute to provision of a suitable ecological niche for the intermediate snail vector. Very low prevalence of urinary schistosomiasis was found in other regions. One district in Western Region1 appeared to have 10% prevalence and was identified as a hot spot for schistosomiasis. *Schistosoma mansoni* (*S*. *mansoni*) was previously assumed to be totally absent in The Gambia, however, this was disproved by our survey. In Central and Upper River Regions, the prevalence of *S*. *mansoni* seemed to be quite negligible at just 0.4% and 0.1% respectively.

The prevalence of soil-transmitted helminthiases nationally indicated The Gambia can achieve elimination quite soon. Of all enteric parasites identified, Ascaris lumbricoides seem to be the most common at 1.8%, hookworm infection was much lower at 0.6% and *Trichuris trichiura* was found to be barely 0.1%. *Ascaris lumbricoides*, was found mainly in Western Region1 where its prevalence appeared to be 4.0%. In Lower River Region, 3.1% of the tests were positive. This relatively low level of prevalence of soil-transmitted helminthiases could be attributed to the contributions made by administration of Mebendazole, being implemented for over two decades by the national immunization program (EPI), in collaboration with National Nutrition Agency’s Vitamin A and Mebendazole supplementation programs. The result further revealed hotspots of *A*. *lumbricoides*. The district of Banjul appeared to be a hot spot for helminthiases because, 55% of the children enrolled tested positive. This is the highest recorded prevalence for *A*. *lumbricoides* in any district in the country. These findings could be attributed to inadequate sanitary facilities and poor drainage in the city to suffice its dense population of both inhabitants, as well as the surging number of people visiting daily for work. These people include businessmen, travelers on transit to the north bank of the River Gambia and beyond, government employees, dockers and other workers at the sea port, the fishing communities, students, and pupils attending schools in Banjul from elsewhere. Hookworm infection, which was found to be low across the country, is concentrated in Western Region 2 where the prevalence was estimated at 2%. We detected *T*. *trichiura* mainly in Western Region 1 where the prevalence seemed to be only 0.5%. Female pupils appeared to be 1.3 times more likely to be infected with the three soil-transmitted helminthiases infections of interest than their male counterparts. This could be explained by the existence of much more risky behavior for STH transmission among female school age children than their male counterparts. Such as proper handwashing with water and soap before eating and after using toilet facilities.

Soil-transmitted helminthiases and schistosomiasis co-endemically exist in 14 (38%) districts. However, for preventive chemotherapy in the case of schistosomiasis and soil-transmitted co-endemic districts, only Praziquantel will be administered using an MDA strategy. The use of Albendazole for treatment of these districts will be on a case-by-case basis according to WHO PC guidelines [17]. The same strategy for Albendazole will be carried out in the remaining STH endemic districts totaling 21 districts or 57% with the exception of two, namely; Banjul and Kombo South. Banjul, with a very high STH prevalence rate of 55%, received two rounds of Albendazole in an MDA in 2017. Kombo South, with a moderate STH prevalence rate of 22%, received one round of treatment with Albendazole in 2017. The remaining 18 SCH endemic districts (equivalent to 49% of the total number of districts) were treated with Praziquantel using varying strategies as recommended by WHO [17]. In SCH endemic districts with low prevalence rate of less than 10%, totaling 13 districts or 72% of districts assessed, all school age children (SAC) enrolled and non-enrolled were treated in a single round of MDA in 2017. Subsequent treatment was planned after 2–3 years. In schistosomiasis endemic districts with moderate prevalence rates of 10–50% (4 districts or 22% of districts assessed), school age children enrolled and non-enrolled were treated in 2017 and in 2019. There will be no repeat rounds of treatment for SCH in any district because no SCH-high prevalent districts were identified in The Gambia according to the data.

Preventive chemotherapy requirements for schistosomiasis and soil-transmitted helminthiases differ according to WHO guidelines, based on the prevalence rates of each condition. Conversely, for schistosomiasis, districts with prevalence of <10%, referred to as schistosomiasis low prevalent districts, amounting to 14 districts were targeted for treatment of all SACs enrolled and non-enrolled twice during their school years [17]. This was complemented by the provision of Praziquantel in health facilities to treat suspected cases. Districts with medium level of prevalence from 10% to 50% amounting to 12 districts will require all SACs enrolled and non-enrolled to be treated once every two years. No SCH endemic district was found to have a prevalence >50%. Only two STH endemic district had a prevalence beyond treatment level on a case-by-case basis. Thus, Kombo South, with medium prevalence, was targeted for treating all SACs and PSACs enrolled and non-enrolled once annually, including the treatment of adults at risk. Banjul district with high prevalence was targeted for two rounds of treatment per year. No mass treatment was conducted in the 19 STH endemic districts with low prevalence rates. See Table 3 above for more details.

**Table 3:** Strategy for Preventive Chemotherapy through MDA implementation in 2017 Fiscal Year based on the status of endemicity at district levels.

Regions	Districts	Endemicity Level		Preventive Chemotherapy (PC)-Strategy			
		Soil-transmitted helminthiasis (sth)	Schistosomiasis (sch)	Albendazole (alb), Only	Praziquantel (pzq), Only	Albendazole + Praziquantel	Remarks
**CRR**	Fulladu West	**1.27%**	**19.45%**				alb = *case; pzq = MDA
**CRR**	Niamina East	**3%**	**5.5%**				alb = case; pzq = MDA
**CRR**	Niamina West	**0%**	**2.5%**				sch = MDA
**CRR**	Niani	**1.6%**	**22.4%**				alb = case; pzq = *MDA
**CRR**	Nianijaa	**0%**	**3.81%**				pzq = MDA
**CRR**	Sami	**0.78%**	**18.24%**				alb = case; pzq = MDA
**LRR**	Jarra Central	**8%**	**1%**				alb = case; pzq = MDA
**LRR**	Jarra East	**2.67%**	**2%**				alb = case; pzq = MDA
**LRR**	Jarra West	**0%**	**2.5%**				pzq = MDA
**LRR**	Kiang Central	**3%**	**1.5%**				alb = case; pzq = MDA
**LRR**	Kiang East	**2%**	**0%**				alb = case
**LRR**	Kiang West	**5.71%**	**0%**				alb = case
**NBE**	Lower Baddibu	**0.8%**	**0.4%**				alb = case; pzq = MDA
**NBE**	Upper Baddibu	**0.82%**	**0.7%**				alb = case; pzq = MDA
**NBW**	Lower Nuimi	**0.29%**	**0%**				alb = case
**URR**	Fulladu East	**1.54%**	**3.99%**				alb = case; pzq = MDA
**URR**	Wuli	**2%**	**19.09%**				alb = case; pzq = MDA
**WR**	Foni Bondali	**6%**	**0%**				alb = case
**WR**	Foni Jarrol	**0%**	**10%**				pzq = MDA
**WR**	Kombo Central	**7.2%**	**0.25%**				alb = case; pzq = MDA
**WR**	Kombo East	**2.2%**	**1.4%**				alb = case; pzq = MDA
**WR**	Kombo North	**3.89%**	**0%**				alb = case
**WR**	Kombo South	**22%**	**0%**				alb = MDA
**WR1**	Banjul	**55%**	**0%**				alb = MDA
**WR1**	Kanifing	**1.27**	**0.07%**				alb = case

KEY: For STH, 0.1–19.9% = Low Prevalence, 20.0–49.9% = Medium Prevalence, ≥ 50% = High Prevalence, For SCH, 0.1–9.9% = Low Prevalence, 10.0–19.9% = Medium Prevalence, 20.0–49.9% = High Prevalence, ≥ 50% = Very High Prevalence, * Case = Treatment on a case-by-case basis, *MDA = mass drug administration, *Rx = Treatment

Despite having a low national prevalence of barely 4%, *S*. *haematobium* is prevalent in Central and Upper River Regions recording regional prevalence of 14.2% and 9.4% respectively. This makes all school age children in the two neighbouring regions eligible for mass treatment with Praziquantel every two years [17]. Concerning the highest prevalence areas of soil-transmitted helminths in the country, Banjul district was prominent with the highest prevalence of 55%. Therefore, two annual rounds of mass treatment would be required using Albendazole.

### Study limitation

One of the limitations in this study was that microscopic diagnosis of intestinal helminths and schistosomiasis has been reported to be associated with low sensitivity especially in communities with light infection rates where parasites might shed fewer eggs, which could compromise the true prevalence of the disease in those communities.

Furthermore, we consider as a limitation this paper, the exclusion of quantitative analysis on intensity of infection or egg counts, as well as, urine dipstick and CCA results. The purpose of the mapping survey was basically to identify the number of endemic districts for schistosomiasis and soil-transmitted helminthiases and to determine their endemicity status to inform plans for control and elimination interventions.

## Conclusion

In conclusion, based on these findings, 24 districts required treatment using mass drug administration strategy, twenty-two of these districts required treatment for schistosomiasis with Praziquantel as implemented in May 2017. The two districts, namely Banjul and Kombo South, were to be treated using Albendazole. Unlike Kombo South, Banjul required two MDA treatment rounds in 2017 due to its very high prevalence of STH.
